# Social network analysis of stakeholder networks from two community-based obesity prevention interventions

**DOI:** 10.1371/journal.pone.0196211

**Published:** 2018-04-27

**Authors:** Jaimie McGlashan, Melanie Nichols, Ariella Korn, Lynne Millar, Jennifer Marks, Andrew Sanigorski, Mark Pachucki, Boyd Swinburn, Steven Allender, Christina Economos

**Affiliations:** 1 Global Obesity Centre (GLOBE), Centre for Population Health Research, Deakin University, Geelong, Australia; 2 Friedman School of Nutrition Science and Policy, Tufts University, Boston, MA, United States of America; 3 Department of Sociology, Computational Social Science Institute, University of Massachusetts, Amherst, MA, United States of America; 4 School of Population Health, University of Auckland, Auckland, New Zealand; University of Sydney, AUSTRALIA

## Abstract

**Introduction:**

Studies of community-based obesity prevention interventions have hypothesized that stakeholder networks are a critical element of effective implementation. This paper presents a quantitative analysis of the interpersonal network structures within a sub-sample of stakeholders from two past successful childhood obesity prevention interventions.

**Methods:**

Participants were recruited from the stakeholder groups (steering committees) of two completed community-based intervention studies, Romp & Chomp (R&C), Australia (2004-2008) and Shape Up Somerville (SUS), USA (2003-2005). Both studies demonstrated significant reductions of overweight and obesity among children. Members of the steering committees were asked to complete a retrospective social network questionnaire using a roster of other committee members and free recall. Each participant was asked to recall the people with whom they discussed issues related to childhood obesity throughout the intervention period, along with providing the closeness and level of influence of each relationship.

**Results:**

Networks were reported by 13 participants from the SUS steering committee and 8 participants from the R&C steering committee. On average, participants nominated 16 contacts with whom they discussed issues related to childhood obesity through the intervention, with approximately half of the relationships described as ‘close’ and 30% as ‘influential’. The ‘discussion’ and ‘close’ networks had high clustering and reciprocity, with ties directed to other steering committee members, and to individuals external to the committee. In contrast, influential ties were more prominently directed internal to the steering committee, with higher network centralization, lower reciprocity and lower clustering.

**Discussion and conclusion:**

Social network analysis provides a method to evaluate the ties within steering committees of community-based obesity prevention interventions. In this study, the network characteristics between a sub-set of stakeholders appeared to be supportive of diffused communication. Future work should prospectively examine stakeholder network structures in a heterogeneous sample of community-based interventions to identify elements most strongly associated with intervention effectiveness.

## Introduction

The drivers of obesity are inherently complex and prevention has proven challenging in the past, yet key examples of success exist and are important to learn from. Community-based, whole-of-system interventions represent a promising approach to preventing childhood obesity [1]. These interventions target multiple factors across multiple settings, in order to achieve system-wide, sustainable change [2]. Several community-based interventions (CBIs) have achieved significant reduction in the body mass index (BMI) of children [1].

Common to the design and implementation of a number of obesity prevention CBIs has been a core community or stakeholder group [3]. These leadership groups (known by many names but often as a ‘steering committee’ (SC)) comprise stakeholders from diverse organizations and sectors, who collaborate to design and implement interventions [4]. SC members are typically identified by their engagement and capacity to impact a problem within their role in a community or organization, such as those from schools, health services or government sectors [5]. SCs work to achieve effective implementation via community outreach, engagement and building public will [6]. Engagement and teamwork of SC members across community networks appear critical to diffuse an intervention, in order to achieve to system-wide change [7]. SCs comprising influential community members or ‘champions’ have been an integral component of successful obesity prevention CBIs, along with the partnerships and relationships developed [8]. Empirical measurement of SC network strength and how their structure may foster diffusion is lacking. Quantifying these SC networks might assist to explain heterogeneity in intervention results, and could be important diagnostics for communities to increase the intervention’s effectiveness.

Social network analysis (SNA) provides methods to quantify the structure of relationships among individuals [9]. Recent work has applied network analysis techniques to explore stakeholder networks in child nutrition programs [10], and at a community-level to evaluate the collaboration among local community groups [11]. A gap remains to retrospectively quantify SC networks from community-based childhood obesity prevention interventions. Such results may provide important insight into the relationships and operation of SC groups within a community and inform more effective implementation [12].

The Shape Up Somerville (SUS) [13] and Romp & Chomp (R&C) [14] CBIs resulted in significant reduction in BMI z-score for children in the intervention areas. Both SUS and R&C investigators retrospectively hypothesized that the SC networks comprising strong partnerships and engagement of community stakeholders were key contributors to the interventions’ results [8] [15] [16].

SUS (2003-2005) was a community-based environmental change intervention in Somerville, MA, USA that targeted the school, home and community settings of early elementary school children [13]. The SC included individuals from schools, food service, community organizations, academia and local health leaders. The ‘on-going group cohesion and consistent leadership’ were regarded as the most critical factors of the intervention for the effectiveness of the intervention [15].

R&C (2004-2008) used a community capacity building approach to improve healthy eating and active play among 12,000 children aged 0 to 5 years in Geelong, Australia [14]. The intervention consisted of multiple changes to environments in early-childhood care and educational settings. It was led by a SC with representation from local government, early childhood settings, health services and academia. R&C stakeholders documented their perceptions of what contributed to the intervention’s positive results, reporting that partnerships and relationships were a critical factor of success; for example, engagement of major community stakeholders [16].

Analysis of the stakeholder networks that played a key role in the design and implementation of these successful interventions may provide insight into the types of relationships and network structures that supported the implementation and dissemination of intervention activities. The objective of this study was to use social network analysis to retrospectively analyze the structure of the networks present within the SCs of two successful childhood obesity CBIs (SUS and R&C).

## Materials and methods

### Data collection and network definition

Data used in this analysis were collected to inform a larger study: Childhood Obesity Modeling for Prevention And Community Transformation (COMPACT), which aims to apply the principles of systems science to understand community-based childhood obesity interventions [17]. Members from both SUS and R&C SCs were invited in 2015 to complete an online survey administered via Qualtrics (Qualtrics, Provo, UT). The study was approved by the Deakin University Human Ethics Advisory Group and the Tufts University Institutional Review Board. Individuals documented in reports and meeting minutes as committee members were eligible participants. Eligible participants were initially contacted to provide informed written consent to being named in the social network survey, and were then sent a survey invitation.

The survey tool used was developed to evaluate stakeholder knowledge and engagement and to elicit social network data from SCs [7]. The survey incorporated a set of questions to assess the presence and nature of professional relationships (‘social ties’) in the SC members’ networks, as they related to childhood obesity prevention in their community.

Participants reported demographic information including gender, affiliation and level of education. To aid recall, contextual information at the time to which the survey refers (political leaders and other local cultural references) was given to prompt the respondent to locate their thinking to the time of the intervention. Along with this, participants were first asked to reflect on the time of the intervention, and write a short description of where they were working, living and any other information regarding their life during that time (these responses were not retained as part of the survey data).

The social network survey asked participants to name up to 20 people with whom they “*discussed issues related to early childhood obesity*” during the intervention; all people identified by this prompt were included in the ‘discussion’ networks—the broadest network definition. Participants could nominate fellow members of the SC, or other contacts external to the committee. Names were generated retrospectively using free recall, supplemented by prompts from a roster of consenting SC participants to aid memory. For each ‘discussion’ tie, participants were asked how ‘close’ they felt the relationship was on a scale from 1 (not close) to 5 (very close). Relationships identified as 4 (close) or 5 (very close) on the Likert scale were used to form the ‘close’ network. Participants were then asked to identify which relationships they found most influential in shaping their understanding of childhood obesity during the years of the intervention. Relationships nominated in this question (as a binary attribute) were used to generate the ‘influence’ networks.

Survey participants, along with all nominated individuals identified in the survey were represented as nodes in the network. Relationships identified between nodes were defined as ties. ‘Discussion’, ‘influence’ and ‘close’ networks were constructed based on the dyadic covariates related to the relationship classification questions.

### Analysis

The networks were analyzed using social network analysis to provide a set of measures to describe the formation of ties within the SC. Analysis of ‘discussion’, ‘influence’ and ‘close’ networks include calculation of: the number of ties; average number of ties nominated per participant (out-degree); and the percent of ties external to the SC compared to the percent of ties within the SC. These measures were then compared across the three types of networks in each CBI to assess similarity between different types of ties. We used graph correlation methods to assess the extent of comparability between the networks. Visual representations of these networks were also produced with fixed node positions to allow comparative visualization between networks.

Among survey participants only, network density, reciprocity of ties, clustering coefficients and centralization were calculated. Network density measures the ties identified within the SC network, as a proportion of the maximum number of potential ties between network members. Reciprocity of ties is the percentage of symmetrical relationships, where two respondents nominate each other for the same type of relationship. The clustering coefficient measures the tendency for a network to form ‘groups of 3’ and is representative of cohesion among group members [18]. In-degree centralization measures the extent to which an individual dominates the network (high centralization), or if relationships are more evenly dispersed through the network (low). Network data were visualized using Gephi [19] and analyzed using igraph [20], a package for R.

## Results

Response rate and network member classification information is shown in Table 1. There were 25 identified SC members from SUS. Of these, 15 consented and thus were named in the survey roster and invited to complete the survey, and 13 completed the survey. In the resulting networks, 19 members (76%) of the SC were included, along with an additional 80 individuals external to the SC. Of the 22 SC members identified from R&C records, 12 were able to be contacted and 8 participated. In the network data gathered from R&C, all 22 SC members (100%) appeared in the network along with 32 individuals external to the SC.

**Table 1:** Node types in the Shape Up Somerville (SUS) and Romp & Chomp (R&C) discussion networks.

Node Classification	SUS	R&C
SC respondents (blue)	13	8
Non-respondent consenting SC members (white)	2	4
Non-respondent non-consenting SC members (grey)	4	10
External to SC (red)	80	32
**Total**	**99**	**54**

Participant demographic information is shown in Table 2. The respondents from R&C were predominantly affiliated with a university (62.5%) and half had a doctorate degree. For the SUS respondents, the most common group affiliations were university (30.8%) and community organizations (30.8%), majority had a masters degree (61.5%), with one respondent having a doctorate degree.

**Table 2:** Demographics and affiliation information of survey participants from Shape Up Somerville (SUS) and Romp & Chomp (R&C).

	SUS *N (%)*	R&C *N (%)*
**Female**	11 (84.6)	5 (62.5)
**Group Affiliation**		
University	4 (30.8)	5 (62.5)
Community-based organization	4 (30.8)	1 (12.5)
School administrator	1 (7.7)	-
After school programs	2 (15.4)	-
Local health department	2 (15.4)	-
Local government	-	2 (25.0)
**Education**		
Diploma or Associate’s degree	-	1 (12.5)
Bachelor’s degree	3 (23.1)	3 (37.5)
Graduate certificate or graduate diploma	1 (7.7)	-
Master’s degree	8 (61.5)	-
Doctorate	1 (7.7)	8 (50.0)

**Fig 1:**
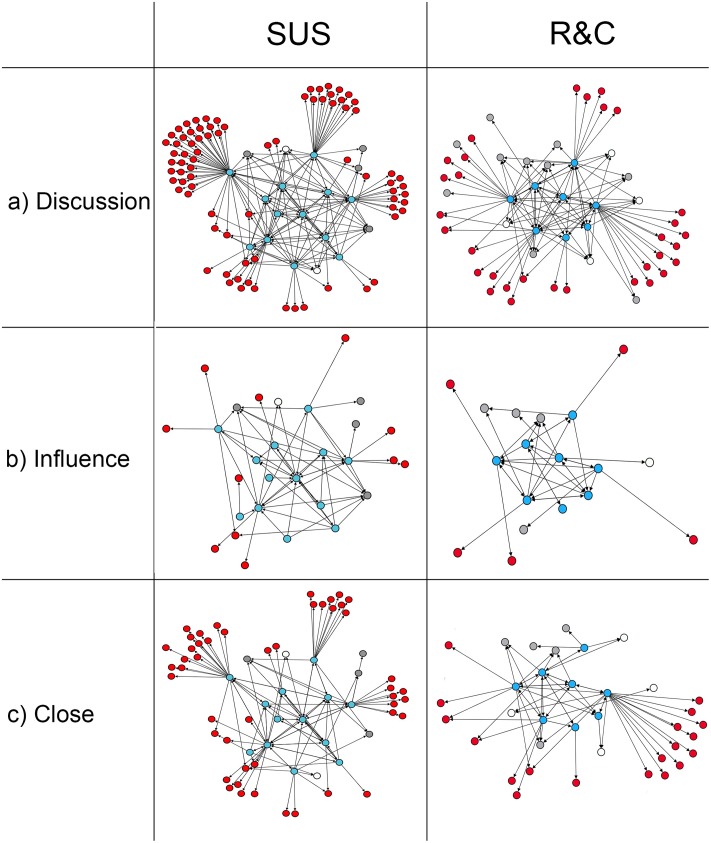
Diagrams of the Shape Up Somerville (SUS) and Romp & Chomp (R&C) steering committee networks for a) discussion, b) influential and c) close relationships during the community-based childhood obesity prevention interventions. Key: Blue = respondents, White = non-respondent consenting SC members, Grey = non-consenting members, and Red = other nominated contacts external to the SC.

Fig 1 shows the (a) ‘discussion’, (b) ‘influence’ and (c) ‘close’ networks for both SUS and R&C. Survey respondents are represented by blue nodes, non-respondent but consenting SC members are white nodes, non-consenting members are grey nodes and other nominated contacts external to the SC are red nodes. When asked to identify people with whom they discussed childhood obesity (‘discussion’ networks), the SUS SC identified 218 relationships among 99 individuals in total (Table 3). Of these, 125 ties (57%) were directed to other members of the SC (Fig 1: blue, grey and white nodes), and 93 (43%) ties were to people outside the SC (Fig 1: red nodes). In the R&C network, a total of 126 discussion ties were identified among 54 individuals, with 90 ties (71%) to members within the SC and 36 (29%) to external contacts.

**Table 3:** Shape Up Somerville (SUS) and Romp and Chomp (R&C) steering committee network metrics.

	Shape Up Somerville (SUS)			Romp & Chomp (R&C)		
	Discussion	Influence	Close	Discussion	Influence	Close
**Ties (n, %)**	218 (100%)	63 (28.9%)	122 (56.0%)	126 (100%)	37 (29.4%)	66 (52.3%)
**Average Out-Degree**	16.8	4.8	9.4	15.8	4.6	8.3
**Within SC (%)**	57.3%	81.0%	56.6%	71.4%	86.5%	66.7%
**Density** ^1^	0.58	0.21	0.35	0.75	0.41	0.45
**Reciprocity (%)** ^1^	73.3%	36.4%	70.4%	81%	52.2%	80.0%
**Clustering (global)** ^1^	0.75	0.45	0.56	0.91	0.70	0.65
**Centralization** ^1^	0.34	0.62	0.40	0.11	0.30	0.27

^1^ Calculated among respondents only (blue nodes in Fig 1)

On average, survey respondents nominated approximately 16 people with whom they discussed issues related to childhood obesity (average out-degree SUS = 16.8; R&C = 15.8) (Table 3). Discussion ties were highly reciprocated between respondents (SUS = 73%; R&C = 81%), as discussion is typically a mutual activity, along with high global clustering (SUS = 0.75; R&C = 0.91), a measure of network cohesion. Centralization was higher in SUS (0.34) reflecting a higher dominance of discussion ties directed to an individual in the network compared to R&C (0.11), where discussion was more evenly dispersed.

Approximately 30% of ties were labelled as influential (SUS = 28.9%; R&C = 29.4%), with SC members identifying five influential relationships on average (out-degree SUS = 4.8; R&C = 4.6) (Fig 1b). Influential relationships were more likely to be directed to other SC members (SUS = 81.0%; R&C = 86.5%), than external to the SC. Analyzing the relationships among survey participants, reciprocity was lowest in influence networks (36.4%; 52.2%), meaning there was a lower likelihood that any pair of participants would both label each other as influential. The influence networks had the highest centralization for both SUS and R&C, meaning that influential advice was likely sought from a single member. This property was more prominent in the SUS influence network, where a single SC member received substantially higher influence ties than others (centralization = 0.62).

In both SUS and R&C, over half of the ties were described as ‘close’ or ‘very close’ relationships (SUS = 56.0%; R&C = 52.3%) (Fig 1c). In contrast to the influential relationships, close relationships were not more substantially directed internal to the SC (SUS = 56.6%; R&C = 66.7%), with respondents reporting many close relationships outside of the SC group. Graph correlation indices showed that in both SUS and R&C, there was a high association between ‘discussion’ and ‘close’ ties. The correlation between ‘influence’ and ‘close’ networks were higher for SUS (similarity = 0.58) compared to R&C (0.35), indicating more alignment between influential and close relationships in the SUS SC (Table 4).

**Table 4:** Graph correlation indices between discussion, influence and close networks between participants for both Shape Up Somerville (SUS) and Romp & Chomp (R&C).

**SUS**	**Discussion**	**Influence**	**Close**	**R&C**	**Discussion**	**Influence**	**Close**
**Discussion**	1.00	-	-	**Discussion**	1.00	-	-
**Influence**	0.44	1.00	-	**Influence**	0.48	1.00	-
**Close**	0.62	0.58	1.00	**Close**	0.52	0.35	1.00

## Discussion

We sought to apply social network analysis to the stakeholder networks from two past childhood obesity prevention interventions. The two interventions both resulted in significant reduction of BMI-z in children, and had each included a steering committee that had been discussed as a contributor to success. The use of social network analysis quantified the structure and strengths of the interpersonal networks between stakeholder members during the intervention period. The subset of stakeholders from SUS and R&C reported similar network structures when asked to retrospectively consider their professional relationships regarding childhood obesity in their communities during the intervention. Although the interventions were designed independently and in different continents, the network structures tended to align and displayed properties such as high clustering and reciprocity.

Among the survey respondents, heterogeneity was observed in the types and structure of relationships. The difference observed between the ‘close’ and ‘influential’ networks (especially noticed in R&C), is not a rare occurrence and has been noted in other contexts, such as social networks within sporting clubs [21]. Relationships labelled as ‘close’, were distributed between other members of the SC, and contacts external to the committee. In contrast, over 80% of influential relationships were directed to SC members. One possible interpretation is that appropriate personnel for influencing the intervention were on the SCs, as community engagement depends on leaders having capacity and influence to facilitate effective intervention implementation [6].

Measuring density of ties among SC members informs the proportion of realized discussion pathways in the committee. In the present study, the high network density shows substantial discussion around childhood obesity within the group, allowing greater pathways for flow of discussion compared with sparse networks [22]. Although higher density can be an indication of a strong network, this should not be assumed [22], as lower densities may be more effective for facilitating diffusion to broader networks [23]. For example, a prior study in a political setting found that relationships expanding beyond the boundary of a central group were more critical than small dense networks for effective reach and collaboration in networks [24]. Thus, the high density within the SC could potentially be a concern for broader diffusion, however, the presence of discussion and close ties external to the SC, should facilitate diffusion to broader parts of a network [25]. As these obesity prevention interventions are known to have been effective in terms of their community engagement, it is possible that this mix of influential leadership networks with close contacts externally supported program dissemination.

Centralization can be measured in SC networks to inform the level to which ties are either dispersed evenly throughout the group or directed to one or few highly centralized individuals. In the present study, high centralization was found in the SUS influence network. This can be interpreted as either a benefit or limitation to the network’s efficiency. Highly centralized networks can be effective for information diffusion; however, overly centralized networks can reduce shared decision making and commitment of non-central members [23]. A study comparing networks of organizations with a focus on promoting physical activity in Colombia and Brazil also found large differences in centralization, with concerns that the more centralized network faced ‘additional barriers’ for successful implementation [26].

Centralization was lower in the R&C network, which should suggest more divested responsibility; however, this has been documented as a project challenge in empirical reports from R&C stakeholders showing frustration with ‘unclear leadership and governance’ [16]. This could be a result of changes to leadership structure during the project, such as the appointments of multiple project coordinators throughout the intervention period [27].

Within the context of obesity prevention CBIs, high centralization of an individual could lead to high reliance on that member, making any effort susceptible to changes in that person’s role, interest or capacity for prevention. This could result in lower shared decision making and limit sustainability if the individual does not have an ongoing role in the project beyond the funded intervention period. Therefore, it is suggested that future CBIs build contingencies into intervention design and SC formation where such potential barriers of over centralized or under centralized networks are acknowledged.

### Strengths and limitations

A key strength of this study was analyzing social network data from two of the world’s first successful childhood obesity interventions. Although studies have suggested the importance and strength of networks in an obesity CBI, this is one of the first to quantify the structure of leadership groups from successful interventions. However, it can not be concluded that the observed properties are responsible for the success of the intervention without more counter examples, assessed either retrospectively or prospectively.

Recall bias was a potential limitation due to the retrospective nature of the study. However, a retrospective study of comparable time frame in the context of childhood neighbourhood recall found results to be valid and reliable, with little evidence of recall bias [28]. Further, to attempt to minimize recall bias, contextual text was added to the survey (i.e. names of government leaders at the time and other cultural references), to allow respondents to localize the timing of relationships relative to then-current events.

It was not possible to obtain data from all SUS and R&C SC members (i.e., the ‘complete networks’) and hence the results are subject to missing data. However, although we could not analyze outgoing ties from non-respondents, the majority of individuals were still represented in the networks through incoming tie nominations (SUS = 76%, R&C = 100%). This meant we were still able to gain some insight regarding the types of networks that existed during the interventions between both respondents and non-respondents.

A large portion of the respondents from the R&C SC were affiliated with the same organization (a university), which could explain the higher percentage of discussion observed within the SC. This result also means SC members who were affiliated with other organizations may be under represented. The absence of non-respondent data may introduce potential bias as it may be that their place in the network led to them being hard to contact in retrospect. Alternately, the occurrence of missing data may be random due to staff turnover and changing contact details between the end of the intervention and the data collection period.

### Questions for future work

CBIs are complex and it is likely intervention success is influenced by, but does not solely depend on, the representation and structure of stakeholder groups. This study reported network characteristics that were present in two successful CBIs. Without larger samples of communities demonstrating heterogeneity of both network structure and intervention effectiveness we cannot make inference about cause and effect, though these generate good hypotheses for future work. Future work comprising retrospective analyses should explore this and include less effective or ineffective interventions to determine whether it can be concluded that network structure is related to success.

### Implications for practice

The analysis of intervention stakeholder networks allows description and comparison of the structure of different types of ties. In this study, discussion, influence and close relationships were analyzed from the networks of two successful childhood obesity interventions. The methods and results from this paper may assist with future CBIs by informing the recruitment of SCs and development of leadership networks in community-based obesity prevention interventions. Future CBI research should include similar analyses prospectively so that the evolution of networks can be captured in real time. These findings can then be used to inform and improve implementation.

## Conclusion

Social network analysis provided insight into the interactions between SC members in relation to childhood obesity throughout two interventions. Future work should prospectively examine network structure in a larger sample of communities to identify the elements of network structure most strongly associated with intervention effectiveness.

## Supporting information

S1 File Network data file. (ZIP)
